# Introduction

**Published:** 2004

**Authors:** Vivian B. Faden, Mark Goldman

**Affiliations:** Co-Leaders, NIAAA Interdisciplinary Team on Underage Drinking Research, October 2005

Underage drinking is a complex problem that has plagued society for generations. As the lead Federal agency for supporting and conducting basic and applied research on alcohol problems, the National Institute on Alcohol Abuse and Alcoholism (NIAAA) has long been at the forefront of efforts to address the broad spectrum of issues related to drinking by youth through projects ranging from studies of alcohol consumption among adolescents to trials of ways to prevent and treat underage drinking.

Over the past decade, NIAAA’s research investment in underage drinking has increased steadily, especially during the 5-year National Institutes of Health budget-doubling period that began in 1998. Increased funding during this period allowed NIAAA to support additional studies and undertake its 1998 college drinking initiative, a collaborative enterprise involving researchers and college presidents.

The college drinking initiative advanced our understanding of drinking by college students, particularly the heavy episodic alcohol consumption commonly called “binge” drinking, and is leading to ways to treat and prevent alcohol-related problems among college students. It also underscored the fact that although some students begin drinking in college, most begin drinking much earlier—in high school, middle school, and even elementary school.

Previously, data from NIAAA’s National Longitudinal Alcohol Epidemiologic Survey (NLAES) had shown that alcohol use early in life correlates strongly with the development of alcohol dependence later in life. In March 2002, NIAAA partnered with the Robert Wood Johnson Foundation to launch the *Leadership to Keep Children Alcohol Free Initiative*. Spearheaded by Governors’ spouses, this initiative focuses on increasing public awareness of alcohol consumption among children ages 9 to 15 and ensuring that policymakers remain fully informed of the seriousness of the childhood drinking issue.

The convergence of a number of recent public events and new scientific developments makes this a particularly opportune and appropriate time to intensify our research, evaluation, and outreach efforts to confront this critical public health issue. First, new findings from NIAAA-supported research indicate that the kind of serious drinking problems previously associated with middle adulthood (including what has been called alcoholism) often begin to emerge during adolescence and young adulthood. These findings, and other results that have enhanced our understanding of alcohol consumption during adolescence, have led to a reconceptualization of alcohol dependence.

We now believe that our science will advance optimally by investigating alcohol-problems in a developmental context. In fact, alcohol abuse and dependence are probably best characterized as developmental disorders, with sequelae that play out throughout the life span. Furthermore, neurobiological research suggests that adolescence may be a period of particular vulnerability to the effects of alcohol.

In addition, the recent National Research Council and Institute of Medicine report, *Reducing Underage Drinking: A Collective Responsibility,* underscores the dangers of underage drinking, even when the level of drinking falls short of a diagnosable condition. This report also proposes a strategy to begin to address this issue. Although we clearly must act now, it also is clear that our currently limited understanding of how alcohol influences adolescent development will limit the success of prevention and intervention efforts. For example, adolescents comprise a very diverse population—a 13-year-old and a 17-year-old differ in many ways, both physically and psychologically. Thus, a single approach for preventing underage drinking will likely be less effective than multiple, developmentally appropriate approaches, even if the primary means for reaching all adolescents is via their parents. To truly understand the risk and protective factors for, and consequences of, alcohol consumption during the first decades of life, we must study alcohol consumption as a developmental phenomenon that begins in childhood and continues through adolescence and into young adulthood.

This issue of *Alcohol Research & Health* is a first step in NIAAA’s efforts to bring the developmental perspective to bear upon the problem of underage drinking. In this special issue, we review the many domains that have been shown by research to interface with alcohol consumption by youth and evaluate the latest research findings in each of these domains. Because a developmental perspective has yet to be fully integrated with research on alcohol and youth, any present review of existing research necessarily must remain limited in this regard. For this reason, since May 2004, NIAAA has been consulting with experts in biological and behavioral development to attempt a significant advance in understanding the phenomenon of underage drinking. To this end, scientists from NIAAA’s staff currently are at work with these developmental scientists to produce a “next generation” report that will explicitly address how developmental science can inform both (a) understanding of the origins of underage drinking and (b) strategic planning to reduce the adverse outcomes suffered by our most precious resource, our children. This new report will, for the first time, explicitly consider the issues involved with underage drinking as a function of age and developmental stage. We anticipate this followup report to be available in the spring of 2006.

In closing, we wish to enthusiastically acknowledge the 2004 report from the National Research Council and the Institute of Medicine (IOM), *Reducing Underage Drinking: A Collective Responsibility.* There are many areas of overlap between the articles in this issue of *Alcohol Research & Health* and the IOM report, and some areas of divergence. In part because of the availability of the earlier report and its extensive accompanying background material, the articles included here could be limited to brief tutorials in many relevant areas of science, and could build upon the earlier contributions by increasing coverage of biological and other laboratory findings. We hope that this summary of recent scientific findings will help researchers and policymakers move forward expeditiously to address the critical problem of underage drinking.

Vivian B. Faden, Ph.D.

Mark Goldman, Ph.D.

Co-Leaders, NIAAA Interdisciplinary Team on Underage Drinking Research, October 2005

